# Childhood Antisocial Behavior and Adolescent Alcohol Use Disorders

**Published:** 2002

**Authors:** Duncan B. Clark, Michael Vanyukov, Jack Cornelius

**Affiliations:** Duncan B. Clark, M.D., Ph.D., and Jack Cornelius, M.D., M.P.H., are both associate professors of psychiatry and pharmaceutical sciences at the University of Pittsburgh School of Medicine and School of Pharmacy, Pittsburgh, Pennsylvania. Michael M. Vanyukov, Ph.D., is assistant professor of pharmaceutical sciences, psychiatry, and human genetics at the University of Pittsburgh School of Medicine and School of Pharmacy, Pittsburgh, Pennsylvania

**Keywords:** comorbidity, childhood behavioral problem, antisocial behavior, adolescent, AODD (alcohol and other drug dependence), alcoholic beverage, conduct disorder, disinhibition, genetic linkage, risk factors, prevention, patient assessment, psychosocial treatment method, literature review

## Abstract

Antisocial behaviors (e.g., aggression toward people and animals, destruction of property, deceitfulness, theft, and serious rule violations) and related mental disorders (i.e., conduct disorder and oppositional defiant disorder) during childhood predict alcohol use disorders (AUDs) during adolescence. This sequence of disorders may reflect developmentally specific forms of deficits in the ability to control behavior. Therefore, childhood antisocial behaviors and adolescent AUDs may share common genetic and environmental influences. A comprehensive conceptual model may clarify the relationship between childhood antisocial behaviors and adolescent AUDs. A better understanding of this relationship is essential for advancing research into the causes of both behaviors and for developing prevention programs and treatment for adolescents with these problems. Prevention programs targeting childhood antisocial behaviors have met with some success. Clinical interventions for adolescents with AUDs may be improved by focusing evaluation and treatment planning on antisocial behavior.

Childhood antisocial behaviors are a central element in the developmental pathway leading dence. Theories and empirical observations indicate that childhood antisocial behaviors increase the risk for alcohol use disorders (AUDs). In its most severe forms, childhood antisocial behavior can lead to diagnoses of conduct disorder (CD) or oppositional defiant disorder (ODD). Particularly for children meeting the criteria for CD, childhood antisocial behaviors predict early initiation of alcohol use, adolescent alcohol-related problems, and the onset of AUDs (Cadoret et al. 1995; Clark et al. 1998*a*, 1999). (Throughout this review, the term “childhood” will refer to age 12 and younger, and “adolescence” will refer to ages 13 through 18.) Understanding the nature of the relationship between antisocial behaviors and AUDs is essential in planning interventions designed to prevent or ameliorate both types of behaviors or disorders.

This article reviews antisocial behaviors and related mental disorders commonly found in children and adolescents and describes the relationship between antisocial behaviors and alcohol problems. The article then presents a conceptual model for explaining this relationship, including genetic and environmental factors that may play a role in the process. Finally, the article summarizes the implications of the relationship between antisocial behaviors and AUDs for understanding the etiology of AUDs, for developing effective methods to prevent alcohol problems, and for evaluating and treating adolescents with AUDs.

## Definitions of Antisocial Behavior and Related Disorders

### Behaviors and Diagnoses

Antisocial behaviors are any acts that violate social rules and the basic rights of others. They include conduct intended to injure people or damage property, illegal behavior, and defiance of generally accepted rules and authority, such as truancy from school. These antisocial behaviors exist along a severity continuum. When childhood antisocial behaviors exceed certain defined thresholds— the diagnostic criteria specified in the *Diagnostic and Statistical Manual of Mental Disorders, Fourth Edition* (DSM–IV) (American Psychiatric Association 1994)—the child is considered to have CD or ODD. Together with attention deficit hyperactivity disorder (ADHD), these two disorders are classified as “disruptive behavior disorders” in the DSM–IV.

#### Conduct Disorder (CD)

Antisocial behaviors represented in the DSM–IV diagnostic criteria for CD include aggression toward people and animals, destruction of property, deceitfulness, theft, and other serious social rule violations (see textbox, below). A diagnosis of CD also requires a persistent behavior pattern in which 3 or more of a total of 15 behaviors occur over a 12-month period. The DSM–IV specifies childhood-onset and adolescent-onset types of CD and different degrees of severity of the disorder.

**Diagnostic Criteria for Conduct Disorder**

Conduct disorder is diagnosed if a persistent pattern of behavior involving three or more of the following behaviors is present over a 12-month period.

**Aggression toward people and animals**

- Often bullies, threatens, or intimidates others
- Often initiates physical fights
- Has used a weapon that can cause serious physical harm to others
- Has been physically cruel to people
- Has been physically cruel to animals
- Has stolen while confronting a victim
- Has forced someone into sexual activity

**Destruction of property**

- Has deliberately set fires with the intention of causing serious damage
- Has deliberately destroyed the property of others

**Deceitfulness or theft**

- Has broken into someone else’s house, building, or car
- Often lies to obtain goods or favors or to avoid obligations
- Has stolen items of nontrivial value without confronting a victim

**Serious violations of rules**

- Often stays out at night despite parental prohibitions, beginning before age 13
- Has run away from home overnight at least twice while living in parental or parental surrogate home
- Often truant from school, beginning before age 13

SOURCE: Adapted from the *Diagnostic and Statistical Manual of Mental Disorders, Fourth Edition* (American Psychiatric Association 1994).

#### Oppositional Defiant Disorder (ODD)

ODD is characterized by negativistic, hostile, and defiant behaviors, such as losing one’s temper, arguing, defying rules, deliberately annoying others, blaming others for one’s behavior, and displaying anger or vindictiveness (see textbox, p. 111). In addition, a diagnosis of ODD according to the DSM–IV criteria requires a pattern of behavior lasting at least 6 months in which 4 or more of a total of 8 behaviors are exhibited. A diagnosis of CD supercedes ODD—that is, if a child meets the criteria for both CD and ODD, he or she will be diagnosed with CD.

**Diagnostic Criteria for Oppositional Defiant Disorder**

Oppositional defiant disorder is diagnosed if a pattern of behavior involving four or more of these criteria is present for at least 6 months.

- Often loses temper
- Often argues with adults
- Often actively defies or refuses to comply with adults’ requests or rules
- Often deliberately annoys people
- Often blames others for his or her mistakes or behavior
- Is often touchy or easily annoyed by others
- Is often angry or resentful
- Is often spiteful or vindictive

SOURCE: Adapted from the *Diagnostic and Statistical Manual of Mental Disorders, Fourth Edition* (American Psychiatric Association 1994).

### Dimensions of Antisocial Behavior

Diagnoses summarize a constellation of characteristics as the presence or absence of a disorder. Although diagnostic classifications of such antisocial behaviors as CD and ODD have both practical and scientific utility, one can also conceptualize these behaviors as occurring along multiple dimensions. Relevant dimensions include the categories of behaviors required for a diagnosis of CD, such as aggression and deceitfulness. One can also distinguish between overt antisocial behaviors, such as fighting, and covert antisocial behaviors, such as theft without confronting the victim (Loeber et al. 2000). The extent to which such dimensions correspond to the diagnostic classifications specified in DSM–IV is a matter of some debate. On the one hand, the available empirical literature indicates that the DSM–IV distinction between ODD and CD is clinically useful for children in general (Loeber et al. 2000) and for adolescents with AUDs in particular (Moss and Lynch 2001). On the other hand, these syndromes are multidimensional, and some features overlap between CD and ODD (i.e., are diagnostically ambiguous) (Hartman et al. 2001). For CD, overt antisocial behaviors may be meaningfully distinguished from covert antisocial behaviors (Loeber et al. 2000).

Developmental considerations are also important for understanding the implications of particular antisocial behaviors for predicting outcomes. For example, the early emergence of aggressive behaviors tends to be accompanied by ODD (Loeber et al. 2000) and to predict later CD (Côté et al. 2001).

### Developmental Continuity and Specificity

Serious antisocial behaviors, including severe forms of ODD and CD, have remarkable developmental stability in boys and girls—that is, these behaviors persist throughout various stages of childhood and adolescence. Mild or moderate forms of the disorders, however, are considerably less stable (Loeber et al. 2000). Antisocial behaviors also tend to be consistent across social settings, such as school and home (Dishion et al. 1995). Although the propensity for serious antisocial behaviors is quite stable across the lifespan, the manifestations of this propensity vary according to developmental stages. This concept has been termed “heterotypic continuity” (Moffitt 1993). For example, antisocial behavior that manifests as irritability and impulsivity in young children may manifest as criminal behavior once these children reach adulthood.

The significance of specific childhood antisocial behaviors also depends, in part, on the timing of their appearance. For example, CD that develops early in life is often preceded by ODD (Loeber et al. 2000), suggesting that ODD behaviors that develop early can predict early onset CD. An earlier age of onset of CD has been hypothesized to indicate more severe antisocial characteristics, although to date empirical support for this hypothesis exists only for boys (Loeber et al. 2000).

The extent to which antisocial behaviors persist across multiple developmental periods also may be an important distinguishing feature (Moffitt 1993). For example, in some people such behaviors occur during childhood, adolescence, and adulthood (i.e., are “life-course persistent”), whereas in other people they are evident only in one developmental stage. This developmental distinction may be useful in understanding the relationship between antisocial behavior and AUDs. Correlations among various antisocial behaviors over time have led to the theory that a general tendency toward psychological dysregulation may underlie many forms of childhood and adolescent psychopathology, including alcohol and other drug use disorders (Tarter et al. 1999).

## Antisocial Behaviors Predict Alcohol Problems

Prospective, longitudinal studies (i.e., studies that followed participants over several years) of children who initially did not exhibit behavior problems have provided clear evidence that childhood antisocial behaviors predict adolescent alcohol involvement and AUDs. Thus, childhood manifestations of deficits in the ability to control behavior (i.e., behavioral undercontrol), including CD and ODD, predict the initiation of regular alcohol use in early adolescence (Clark et al. 1998*a*) and the onset of alcohol-related problems (Clark et al. 1999) and AUDs (Caspi et al. 1996; Rydelius 1981) during adolescence. ADHD may be less relevant because it did not predict AUDs in some studies (Mannuzza et al. 1998). In other studies, ADHD did predict adolescent alcohol and drug problems; however, that association may have been attributable to CD co-occurring in the children with ADHD (Clark et al. 1999). Finally, children of parents with alcohol and other drug use disorders (i.e., high-risk children) have increased rates of antisocial behaviors. Childhood antisocial behavior, such as noncompliance with parental directives in the toddler years (Eiden et al. 2001), and CD and ODD in the school-age years (Clark et al. 1997*a*) are more common in children at high risk for alcohol and other drug use disorders.

Based on these observations, it is clear that childhood antisocial behavior precedes and predicts adolescent AUDs. Consequently, a conceptual model is needed to guide further investigation into the causal relationships between both types of behaviors. Such a model is presented in the following section.

### A Conceptual Model

Conceptual approaches from several traditions have proven useful for developing theories about the relationship between childhood antisocial behaviors and adolescent AUDs. The model presented here, and described in more detail in Clark and Winters (in press), represents an integrated conceptual model and measurement approach that allows researchers to consider the multiple causes and effects shaping this relationship. This model is informed by prior theories (Zucker et al. 1995; Tarter et al. 1999), assessment methodologies (Clark et al. 2001), and research (Clark et al. 1999) in this area. The model combines two approaches:

- *The multifactorial model of complex traits.* This model assumes that individual differences in observable characteristics— in this case, antisocial behaviors and AUDs—are determined by variations in the combined influences of multiple genes and environmental factors (Lander and Schork 1994; Vanyukov and Tarter 2000).
- *The theoretical framework of developmental psychopathology.* This framework emphasizes specific methodological approaches and conceptual issues by contrasting normal and atypical development. It also takes into consideration that the effects of risk factors may vary across developmental stages (Cicchetti and Cohen 1995).

This model, as well as conceptualizations from several other traditions, hypothesizes that childhood antisocial behaviors and adolescent AUD have common causes. Several mechanisms may underlie these common causes. First, both antisocial behaviors and AUDs may be manifestations of a fundamental deficiency in the person’s ability to control or regulate his or her behavior (Tarter et al. 1999). Second, the observed relationship between antisocial behaviors and AUDs may reflect the presence of common genetic factors and/or environmental influences. These mechanisms, which are not mutually exclusive and can both be included within the proposed comprehensive model, are discussed in more detail in the following sections.

### The Dysregulation Hypothesis

A common underlying factor—namely, a tendency toward poor behavioral regulation—may predispose some people to both childhood antisocial behaviors and AUDs (Cadoret et al. 1995). Behavioral undercontrol (also referred to as “behavioral dysregulation” and “disinhibition”) is characterized by deficits in the planning and execution of goal-directed behavior, and is manifested by aggressive, antisocial, and impulsive behavior (Martin et al. 2000), all of which predict problematic alcohol use (Caspi et al. 1996). Behavioral under-control also has been hypothesized to underlie the observed associations among childhood CD, alcohol and other drug use disorders, and adult antisocial personality disorders.

During a person’s development, the ability to regulate and control behaviors and emotions emerges at the same time that a brain region called the prefrontal cortex matures. Accordingly, researchers have hypothesized that the neurobiological functions that modulate thoughts (i.e., cognition), the emotions associated with those thoughts (i.e., affect), and behavior are located in the prefrontal cortex (Spear 2000). Consistent with this hypothesis, neuroimaging findings indicate that abnormalities in the structure of the prefrontal cortex are associated with severe antisocial behavior (Raine et al. 2000). The rate with which certain brain circuits involving the prefrontal cortex mature may be an important mechanism through which genetic factors influence psychopathological manifestations (Todd et al. 1995).

### Genetic Influences

#### Behavior Genetics

Researchers have begun to investigate the extent to which similarities in antisocial behavior and AUDs among relatives result from genetic inheritance (i.e., shared genes) or environmental factors. Studies in this area have provided convincing evidence that genetic factors contribute substantially to individual variations in both antisocial behavior and AUDs (Tarter et al. 1999). Some studies have also suggested that the high correlations between ODD and CD symptoms can be attributed to genetic similarity (Eaves et al. 2000). The characteristic features of behavioral undercontrol are highly susceptible to genetic influence, and common genetic factors may account for the associations between antisocial behaviors and drug use (Young et al. 2000). To explore the role of genetic factors in the intergenerational transmission of antisocial behavior, Cadoret and colleagues (1995) studied adopted children and their biological and adoptive parents. They found evidence for a genetically transmitted pathway leading from antisocial personality disorder and drug use disorders in the biological parent to CD in the offspring and, subsequently, drug use disorders and antisocial personality disorder in the offspring.

Other studies found that the correlation between childhood antisocial behavior and adult drug use disorders is more strongly influenced by genetic factors than is the correlation between adult antisocial behaviors and drug use disorders (Grove et al. 1990). This observation reinforces the notion that childhood characteristics are of fundamental importance for the development of adult behaviors. The relationship between childhood antisocial behavior and the later development of AUDs may be the result of common genetic influences (Waldman and Slutske 2000).

#### Molecular Genetics

In general, variations in the structure of certain genes (i.e., genetic polymorphisms) account for the inheritance of individual differences in behavior. Although extensive evidence has established that heritable factors are a major influence in the development of AUDs, researchers have not yet been able to identify the mechanisms leading to the development of AUDs and the specific genes involved. One candidate that has been implicated in AUDs is a brain signaling system called the dopamine neurotransmitter system. Individual differences in this system are likely to influence the extent to which a person experiences alcohol’s effects as pleasant and therefore wants to consume more alcohol (i.e., the extent to which a person experiences drinking as positively reinforcing). Variations in the level of reinforcement obviously can influence a person’s risk for alcohol and other drug use disorders. Accordingly, researchers have begun to study associations between genetic polymorphisms influencing dopamine and other brain signaling systems on the one hand, and the risk for AUDs on the other hand (Vanyukov and Tarter 2000). These associations may provide insights into the genetic, biochemical, and neurobiological mechanisms underlying AUDs and may also reveal the nature of the relationship between AUDs and antisocial behaviors (Vanyukov et al. 2000).

### Environmental Influences

Several environmental factors have been found to increase the risk for antisocial behavior as well as AUDs in adolescents. These influences include problematic family functioning, such as low levels of parental monitoring and inconsistent disciplinary practices (Clark et al. 1998*b*), as well as childhood maltreatment (Clark et al. 1997*b*). The developmental psychopathology framework described earlier suggests that specific environmental factors may be particularly influential during critical developmental periods. For example, paternal drug use disorders may have differential effects depending on a child’s stage of development. In a study examining psychopathology in a sample of high-risk boys (Moss et al. 1997), boys whose fathers overcame drug use disorders prior to the child’s school-age period were similar to a control group of boys whose fathers had no drug use disorders. Conversely, boys whose fathers had continuing drug use disorders exhibited significant psychopathology.

Parents’ drug use and other pathology may affect the development of their offspring through several mechanisms. For example, parental drug use and other pathology may directly influence parenting behaviors. In addition, the effects of parental pathology may be indirect. Thus, parental mental disorders may act as barriers impeding their children’s access to adequate mental health treatment, which in turn may increase the children’s likelihood of developing AUDs in adolescence (Cornelius et al. 2001).

Environmental influences invariably interact with genetic factors to determine a person’s risk for certain disorders. The dynamic interaction of genetic and environmental influences with certain behaviors over the course of development is particularly complex and therefore difficult to analyze (Tarter et al. 1999). For example, for AUDs to develop, alcohol availability in the environment (e.g., from family or friends) is a necessary but not sufficient condition. An adolescent who chooses peers who use alcohol and other drugs may be more frequently exposed to alcohol than is an adolescent with a different peer group. When combined with a genetic predisposition to alcohol dependence, the adolescent’s selection of deviant peers and consequent high exposure to alcohol may result in the development of AUDs. Such interactions are ideally taken into consideration when studying the relationship between childhood antisocial behaviors and AUDs.

## Implications for Prevention, Evaluation, and Treatment

A causal model explaining the association between childhood antisocial behaviors and the development of AUDs during adolescence, if it can be validated, has implications for the prevention, evaluation, and treatment of those behaviors. For example, early intervention for antisocial behaviors might reduce the risk of developing an AUD, and treatment for AUDs might be more effective if it also addressed behavioral undercontrol. These possible implications are reviewed in the following sections.

### Prevention

A potentially effective strategy to prevent the development of AUDs involves using interventions designed to reduce childhood characteristics that predict adolescent AUDs, such as childhood antisocial behavior. Several research programs have been investigating this approach, and early reports have shown promising results. For example, Linking the Interests of Family and Teachers (LIFT) is an elementary-school prevention program that uses behavior modification with children on the playground, provides social and problem-solving skills training to children in the classroom, and offers parenting skills training to their parents (Eddy et al. 2000). When the children receiving the LIFT intervention and a group of control children were reevaluated 3 years after the intervention, the LIFT program was found to delay the time to first regular alcohol use, first marijuana use, and first police arrest. Other similar projects are in progress (e.g., Ialongo et al. 2001). Researchers also have identified effective interventions for childhood CD (Sheldrick et al. 2001), which may reduce the risk for adolescent AUDs.

### Evaluation

A comprehensive assessment, including a systematic evaluation of the patient’s history of antisocial behaviors and drug use disorders, is the foundation for effective treatment planning for adolescents with these problems. Too often, clinical assessments lack this foundation. For example, clinical assessment strategies are typically unstructured and may lead to inaccurate diagnoses (Clark et al. 1995). Systematic diagnostic interviews provide for a more thorough assessment with greater reliability and validity and have been advocated for both clinical evaluations and research (Clark et al. 1999). Moreover, additional domains should be included in such comprehensive assessments, including parent-adolescent relationships, peer characteristics, school functioning, and health. Clark and Winters (in press) have proposed assessment strategies that are designed to provide the comprehensive and developmentally appropriate information that is necessary for clinical interventions, prevention, and related research.

It is also important to recognize that AUDs have risk factors and consequences that manifest differently at various developmental stages. Assessment of relationships among various behaviors over time requires specialized measurement approaches called “diachronic assessment” strategies (Clark et al. 2001). Recently developed statistical techniques that can provide more realistic summaries of growth and development (e.g., Muthén and Muthén 2000) allow optimal statistical modeling of data obtained with such assessment approaches. The use of diachronic assessment strategies in combination with such innovative statistical modeling techniques can allow insights into the relationships among problem behaviors such as antisocial behavior and AUDs.

### Treatment

Although achieving abstinence from alcohol is the optimal treatment goal for patients with AUDs, adolescents participating in conventional alcoholism treatments have high relapse rates (Cornelius et al. in press). Several clinical studies have indicated that co-occurring CD predicts particularly poor outcomes among adolescents receiving treatment for alcohol and other drug problems (Brown et al. 1996; Crowley et al. 1998; Kaminer et al. 1992). Accordingly, programs for adolescents with AUDs may need to include interventions designed to reduce antisocial behaviors.

Several treatment approaches specifically target CD. Psychosocial interventions with standardized methods and documented effectiveness include training parents in child management techniques and teaching children prosocial responses to interpersonal conflicts (Sheldrick et al. 2001). Stimulant medications, such as methylphenidate (Ritalin^®^) may also improve CD (Klein et al. 1997). The extent to which such psychosocial and pharmacological treatments for CD also improve the outcome of adolescents with coexisting CD and AUDs requires further research.

For patients with co-occurring antisocial behaviors and AUDs, behavioral treatments may be more effective when the interventions target multiple domains, including the individual, family, and peers. Interventions using this strategy, such as the Multisystemic Treatment approach, have been shown to improve outcome compared with less intensive approaches. For example, in a clinical adolescent sample, the Multisystemic Treatment approach reduced both drug use and antisocial behavior (Henggeler et al. 1998).

## Future Directions

Although researchers and clinicians have long recognized the relationship between childhood antisocial behavior and adolescent AUDs, a need remains for further research into the mechanisms underlying this relationship, as well as for prevention and treatment research. For example, treatment studies need to define the cost-effectiveness of interventions and determine how treatment gains made in supervised settings (i.e., clinical studies) can be transferred to real-life settings (e.g., home and school). Moreover, the potential benefits of simultaneously treating antisocial behavior and AUDs must be elucidated further. Future studies also must consider gender differences more thoroughly, because although antisocial behaviors and AUDs are more common in males, females with these characteristics may have more problematic outcomes (Loeber et al. 2000). Finally, as researchers more clearly identify the genetic and environmental influences on childhood antisocial behaviors and adolescent AUDs, they also need to further examine the effects of environmental influences on the persistence of these behaviors.

Treatment programs simultaneously addressing multiple domains represent an ideal and necessary approach for some adolescents with AUDs. The high costs of such comprehensive programs, however, limit their application. Furthermore, many adolescents with AUDs may have acceptable outcomes with conventional, less intensive interventions (Maisto et al. 2001). Therefore, researchers and clinicians must develop more extensive empirical data to serve as a basis for making specific treatment recommendations in order to increase the likelihood that policymakers and payers (e.g., insurance companies) accept the increased costs associated with more comprehensive services. And although the cost of providing intensive interventions for adolescents with AUDs is considerable, the societal cost of neglecting these highly problematic adolescents is even greater (Scott et al. 2001).

Substantial challenges remain in understanding the relationship between childhood antisocial behavior and adolescent AUDs. Both antisocial behaviors and AUDs are complex problems with multiple contributing factors. Consequently, genetic, family, epidemiological, and clinical studies are needed to define clinically meaningful patient subgroups, identify children at highest risk for AUDs, and inform more effective prevention and treatment efforts.
